# Case of Concussion of the Brain

**Published:** 1823-12

**Authors:** Geo. Shipman


Art. III.—*Case of Concussion of the Brain.* By Geo. Shipman, Esq. [Communicated through Dr. James Johnson.]

A case of concussion of the brain has recently come under my immediate observation, presenting peculiarities which I believe are not generally observed in such injuries: a brief outline of

It, therefore, may not prove wholly useless to some of your readers.

The patient is a youth, about eighteen, (my apprentice,) who, on Tuesday morning, the 7th instant, struck his head, on the middle of the anterior edge of the right parietal bone, against a brass nob, but did not wound the scalp. The pain at the time was considerable, but the accident, for some hours afterwards, would have been wholly disregarded, had not sickness and vomiting come on. About four, the same day, he complained of great pain and heat in the part, accompanied with pricking sensations beneath the bone; at nine, he became delirious, pupils contracted, with every other marked symptom of inflammation. About twenty ounces of blood were taken: pain considerably abated. A purging mixture, consisting of infusion of senna, salts, and manna, had been given at intervals of two hours, which had operated twice or thrice. At two the next morning delirium returned, and with it a high degree of nervous excitability; the hearing exceedingly acute. The head was shaved, and twenty leeches applied. Great relief was afforded, and he became again more sensible. Spirit of wine and æther was used as a local application. He continued, during the forepart of the day more tranquil; at five P.M., however, every symptom increased. He was bled to the extent of sixteen ounces: no abatement. The alimentary secretions had not been discharged for some hours: half a grain of calomel, with five of jalap, were given every two hours. Free evacuations were procured, and he was relieved.

On the following morning, however, (the 8th,) they ceased; he refused all medicine. About two P.M. he became blind, deaf, and unable to speak; but, with these symptoms, he was sensible: he rubbed his eyes, and appeared conscious of being blind; was hysterical, sometimes crying, and at others convulsed; made signs to write, and, on presenting him with pen and paper, he wrote down what he wished; then made signs for something to eat. On giving him bread and butter, he ate it voraciously, on which I previously put four grains of calomel. His bowels acted copiously about two hours afterwards, when he almost spontaneously recovered his sight, hearing, &c. From that total want of nervous susceptibility, there was a change to a state of excitement and acute sensibility to all impressions, accompanied with incoherent ideas and expressions, violently resisting every attempt to administer either medicine or food.

On the morning of the 9th, he again became blind, with the same train of symptoms as on the preceding day, but more convulsed. From twenty to thirty leeches were again applied, but no evident amendment. Injections were given, but did not produce satisfactory discharges. As on the former day, he made signs for something to eat, when I again gave him calomel on bread and butter; which having operated two or three times, his speech returned, and he expressed himself as being better, and that he "could see." He continued occasionally convulsed through the day, accompanied with great anxiety and watchfulness. He did not refuse medicine, and discharges from the bowels were regularly kept up, when a progressive amendment was observable; and now (the 14th,) he is sitting up, complaining of little else than debility.

At the time he became blind, the pupils of the eyes were dilated, and unvaried by holding the candle near them. I was at a loss to conceive whether that state might be ascribed to effusion or extravasation: his appearance and actions bore closely on the former, but remaining sensible did away with such a conclusion. The instantaneous change, also, from a state of morbid sensibility of light and sound, to that of a total want of it, rendered it extremely improbable that organic changes were taking place. It was remarked that this state came on after a lapse of five or six hours of inaction of the bowels, and shortly after disappeared when copious secretions were procured. I am decidedly of opinion that, after the second bleeding, inflammation and other consequences of the injury, as respects the *brain*, were removed, and that the subsequent changes were produced by extensive disorder of the stomach, which such injuries are calculated to produce. During the whole of the period, acute pain was experienced upon the slightest pressure over the epigastric region, and the tenderness was observed to be increased during a state of paralyses of the optic and auditory nerves.

Mr. Abernethy visited the case first on the 8th, and daily until the patient's recovery. I could wish to be favoured with some remarks from the pen of that gentleman on the leading points of this case.

*2, Guilford-street, Russell-square; Oct. 14th,* 1823.

